# Splitting an Arbitrary Three-Qubit State via a Five-Qubit Cluster State and a Bell State

**DOI:** 10.3390/e24030381

**Published:** 2022-03-08

**Authors:** Gang Xu, Tianai Zhou, Xiu-Bo Chen, Xiaojun Wang

**Affiliations:** 1 School of Information Science and Technology, North China University of Technology, Beijing 100144, China; 2 Advanced Cryptography and System Security Key Laboratory of Sichuan Province, Chengdu 610025, China; 3 Information Security Center, State Key Laboratory of Networking and Switching Technology, Beijing University of Posts and Telecommunications, Beijing 100876, China; 4 School of Electronic Engineering, Dublin City University, D09 W6Y4 Dublin, Ireland; xiaojun.wang@dcu.ie

**Keywords:** quantum information splitting, bell basis measurement, cluster state, single-qubit measurement

## Abstract

Quantum information splitting (QIS) provides an idea for transmitting the quantum state through a classical channel and a preshared quantum entanglement resource. This paper presents a new scheme for QIS based on a five-qubit cluster state and a Bell state. In this scheme, the sender transmits the unknown three-qubit secret state to two agents by the quantum channel with the Bell basis measurement three times and broadcasts the measurement results to the agents through the classical channel. The agent who restores the secret state can successfully recover the initial information to be transmitted through the appropriate unitary operation with the help of the other party. Firstly, our scheme’s process can be accurately realized by performing the applicable Bell basis measurement, single-qubit measurement, and local unitary operation instead of a multiparticle joint measurement. The splitting process of quantum information is realized through a convenient operation. Secondly, compared with some previous schemes, the efficiency of the total scheme has been improved in principle, and the qubit consumption is reduced. Finally, the security of the quantum information splitting scheme is analyzed from the perspectives of external attacks and participant attacks. It is proved that our scheme can effectively resist internal participant attacks and external eavesdropper attacks.

## 1. Introduction

The traditional public-key cryptography is based on mathematically difficult problems. Moreover, the emergence and development of quantum computers significantly threaten the conventional cryptosystem’s security. However, with the in-depth study of the quantum mechanical phenomena and quantum information theory, security can be guaranteed in the process of encryption technology [1,2] and quantum communication [3,4,5]. Quantum communication, an essential branch of quantum information theory, uses quantum entanglement for quantum transmission. Quantum entanglement exerts an immense influence on the principles of quantum mechanics. It is widely used in quantum communication technologies, such as quantum teleportation [6,7], controlled teleportation [8,9,10], quantum secret sharing (QSS) [11,12,13], and quantum information splitting [14,15]. In general, QSS mainly involves two principles: one is to share classical secret information [16,17], and the other is to share confidential quantum information [18,19]. 

In 1993, Bennett et al. [20] proposed the scheme of quantum teleportation for two-level systems to transmit an arbitrary qubit from the sender to a long-distance receiver. In 1999, the concept of quantum information splitting by extending the idea of Bennett [20] was proposed by Hillery et al. [21] for the first time. Any receiver cannot reconfigure the original state alone but must be assisted by other agents. Quantum information splitting uses quantum entanglement resources to split the transmitted state [22], which can be regarded as the quantum counterpart of classical secret sharing. Since then, quantum information splitting has been extensively studied in theory [23,24] and experiments [25,26]. Subsequently, the splitting schemes for an arbitrary single-qubit secret state [27,28] and two-qubit secret state [29,30,31] have been introduced one after another. Nie et al. [32] designed a deterministic protocol for splitting an arbitrary three-qubit state using a genuinely entangled five-qubit state and a Bell state as the quantum entanglement resource. Shortly afterwards, Zhong et al. [33] demonstrated how to draw support from a four-qubit cluster state and a Greenberger−Horne−Zeilinge state to split the state to be transmitted in the sender’s hand.

In this paper, a new scheme to realize the splitting process of an arbitrary three-qubit state using a five-qubit cluster state and a Bell state as the quantum entanglement resource is presented. Firstly, the sender, Alison, needs to perform the corresponding Bell basis measurements on the three pairs of qubits she owns and send out the measurement outcomes to Bond and Calvin through the classical channel. Secondly, if Bond intends to reconstruct the initial secret information, the assistance of a third-party controller, Calvin, is necessary. It means that Calvin has to performthe *X* basis measurement on his particle and tell Bond the measurement outcome. Finally, by combining the information from Alison and Calvin, Bond can perform the appropriate unitary operations on the owned qubits to reconstruct the state to be transmitted. Compared with the previous schemes, our quantum information splitting process can be completed only by the Bell basis measurement, *X* basis measurement, and simple unitary transformation, without a multiparticle joint measurement. A more convenient way can accomplish the communication process, improve the qubit efficiency and the scheme’s security, and reduce communication costs.

The structure of thispaper is as follows. Section 2 briefly introduces some basic quantum gates and the preparation of the five-qubit cluster state from the principles of quantum circuits. Then, in Section 3, the scheme for splitting an arbitrary three-qubit state using a five-qubit cluster state and a Bell state as the quantum entanglement resource is proposed. Section 4 analyses the scheme’s security from internal participant and external eavesdropper attacks and the efficiency of our scheme. Finally, Section 5 concludes the paper.

## 2. Preliminaries

### 2.1. Basic Quantum Gates

Unitary operators play a significant part in quantum communication and quantum computation. The operators can act on qubits, in which the quantum gate can transform an input qubit state into a desired output state. A set of basic operations with single-qubit states is known as Pauli operators or *I*, *X*, *Y*, *Z* gates. The matrix specific forms and actions on the computational basis states are defined as follows:

(1) $$\begin{array}{l} I=\left(\begin{array}{cc} 1 & 0\\ 0 & 1 \end{array}\right),\;X=\left(\begin{array}{cc} 0 & 1\\ 1 & 0 \end{array}\right),\;\\ iY=i\left(\begin{array}{cc} 0 & -i\\ i & 0 \end{array}\right)=\left(\begin{array}{cc} 0 & 1\\ -1 & 0 \end{array}\right),\;iY|0\rangle =-|1\rangle ,iY|1\rangle =|0\rangle ,\\ Z=\left(\begin{array}{cc} 1 & 0\\ 0 & -1 \end{array}\right) \end{array}$$

The Hadamard matrix gate, or *H* gate, is defined as follows:

(2) $$H\;=\;\frac{1}{\sqrt{2}}\left(\begin{array}{cc} 1 & 1\\ 1 & -1 \end{array}\right)$$

The elementary gate also contains the two-qubit gate, except the single-qubit gates mentioned above, namely the Controlled-NOT (*CNOT*) gate, which is defined as follows:

(3) $$CNOT\;=\;\left(\begin{array}{cccc} 1 & 0 & 0 & 0\\ 0 & 1 & 0 & 0\\ 0 & 0 & 0 & 1\\ 0 & 0 & 1 & 0 \end{array}\right)$$

### 2.2. Cluster State

In Ref. [34], an important kind of entangled states, namely cluster states, was introduced. Cluster states enjoy the following remarkable property: each pair of qubits can be projected into a maximally entangled state with certainty by single-qubit measurements on all the other qubits. This property might suitably be referred to as maximal connectedness.

For conceptual simplicity, we first restrict ourselves to one-dimensional lattices. Let us recall the definition of one-dimensional cluster states for *N* qubits. Consider an *N*-site lattice, with a qubit attached to each site. As a novel multi-qubit entangled state, the cluster state is written as follows:

(4) $${|\phi \rangle }_{C}\;=\;\frac{1}{2^{N/2}}\overset{N}{\underset{a\;=\;1}{⊗}}[{|0\rangle }_{a}\;+\;{|1\rangle }_{a}{\left(\sigma_{z}\right)}_{a\;+\;1}]$$

where ${\left(\sigma_{i}\right)}_{a}$(i = x,y,z) are the Pauli matrices assigned for site a in the lattice, and $\sigma_{z}{|s\rangle }_{a}\;=\;{\left(-1\right)}^{s}|s\rangle$,(s∈{0, 1}).

### 2.3. The Preparation of the Five-Qubit Cluster State

**Figure 1:**
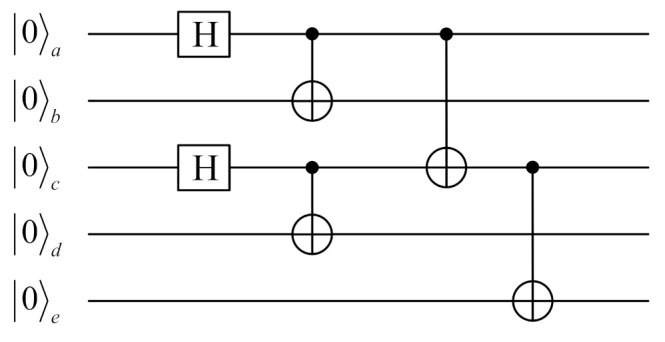
Circuit diagram representation for the preparation of the five-qubit cluster state.

In this section, the quantum circuit that the five-qubit cluster state prepared from five individual single qubits is plotted, which is shown in the following Figure 1.

(1) Prepare the five individual single qubits $\left(a,b,c,d,e\right)$ in the states ${|0\rangle }_{a}$, ${|0\rangle }_{b}$, ${|0\rangle }_{c}$, ${|0\rangle }_{d}$, ${|0\rangle }_{e}$, respectively.
(2) Perform *H* gateon qubits a, c.
(3) Carry out the *CNOT* gate on the qubit pairs $\left(a,b\right)$, $\left(c,d\right)$, $\left(a,c\right)$, and $\left(c,e\right)$, in which the control qubit is the former particle in the qubit pairs, and the target qubitis the second inputparticle, respectively.

Next, the preparation process of a five-qubit cluster state from five qubits can be shown as follows:

(5) $$\begin{array}{l} \;\;\;\;\;{|0\rangle }_{a}⊗{|0\rangle }_{b}⊗{|0\rangle }_{c}⊗{|0\rangle }_{d}⊗{|0\rangle }_{e}\\ \overset{H_{a}⊗I_{b}⊗H_{c}⊗I_{d}⊗I_{e}}{\to }\frac{1}{\sqrt{2}}{\left(|0\rangle +|1\rangle \right)}_{a}⊗{|0\rangle }_{b}⊗\frac{1}{\sqrt{2}}{\left(|0\rangle \;+\;|1\rangle \right)}_{c}⊗{|0\rangle }_{d}⊗{|0\rangle }_{e}\\ \;\;\overset{CNOT_{ab}}{\to }\frac{1}{2}{\left(|00\rangle \;+\;|11\rangle \right)}_{ab}⊗{\left(|0\rangle \;+\;|1\rangle \right)}_{c}⊗{|0\rangle }_{d}⊗{|0\rangle }_{e}\\ \;\;\overset{CNOT_{cd}}{\to }\frac{1}{2}{\left(|00\rangle \;+\;|11\rangle \right)}_{ab}⊗{\left(|00\rangle \;+\;|11\rangle \right)}_{cd}⊗{|0\rangle }_{e}\\ \;\;\overset{CNOT_{ac}}{\to }\frac{1}{2}{\left(|0000\rangle +|0011\rangle \;+\;|1110\rangle \;+\;|1101\rangle \right)}_{abcd}⊗{|0\rangle }_{e}\\ \;\;\overset{CNOT_{ce}}{\to }\frac{1}{2}{\left(|00000\rangle \;+\;|00111\rangle \;+\;|11101\rangle \;+\;|11010\rangle \right)}_{abcde} \end{array}$$

## 3. Splitting an Arbitrary Three-Qubit State

Our scheme is illustrated in detail in this section. There are only three legal parties in this scheme: Alison, Bond, and Calvin. Alisonis the sender; Bond and Calvin are the two agents of Alison. Bond is the information receiver, and Calvin is the controller. Alison first prepares the required quantum entanglement resources as the quantum channel [18], which is given as follows:

(6) $$\begin{array}{l} {|\Psi \rangle }_{12345}\;=\;\frac{1}{2}{\left(|00000\rangle \;+\;|00111\rangle \;+\;|11101\rangle \;+\;|11010\rangle \right)}_{12345}\\ {|\phi^{+}\rangle }_{67}\;=\;\frac{1}{\sqrt{2}}{\left(|00\rangle \;+\;|11\rangle \right)}_{67} \end{array}$$

Thus, the quantum resource of the seven-qubit can be expressed as the following equation:

(7) $$\begin{array}{ll} {|\Theta \rangle }_{1234567} & ={|\psi \rangle }_{12345}⊗{|\phi^{+}\rangle }_{67}\\ & =\frac{1}{2}{\left(|00000\rangle \;+\;|00111\rangle \;+\;|11101\rangle \;+\;|11010\rangle \right)}_{12345}⊗\frac{1}{\sqrt{2}}{\left(|00\rangle \;+\;|11\rangle \right)}_{67}\\ & =\frac{1}{2\sqrt{2}}\left(|0000000\rangle \;+\;|0011100\rangle \;+\;|1110100\rangle \;+\;|1101000\rangle \right.\\ & +{\left.|0000011\rangle \;+\;|0011111\rangle \;+\;|1110111\rangle \;+\;|1101011\rangle \right)}_{1234567} \end{array}$$

Alison possesses an arbitrary unknown three-qubit state as follows:

(8) $$\begin{array}{ll} {|\psi \rangle }_{xyz} & =\left(\delta_{000}|000\rangle \;+\;\delta_{001}|001\rangle \;+\;\delta_{010}|010\rangle \;+\;\delta_{011}|011\rangle \right.\\ & {\left.+\delta_{100}|100\rangle \;+\;\delta_{101}|101\rangle \;+\;\delta_{110}|110\rangle \;+\;\delta_{111}|111\rangle \right)}_{xyz} \end{array}$$

where the three particles *x*, *y*, and *z* make up the state ${|\psi \rangle }_{xyz}$,and each of these coefficients is a complex number that satisfies the condition of normalization.

(9) $${\sum_{l_{1},l_{2},l_{3}\;=\;0}^{1}\left|\delta_{l_{1}l_{2}l_{3}}\right|}^{2}\;=\;1$$

Hence, the entire system can be combined and written as the following state:

(10) $${|\Psi \rangle }_{xyz1234567}\;=\;{|\psi \rangle }_{xyz}⊗{|\Theta \rangle }_{1234567}$$

where Alison owns the particles *x*, *y*, *z*, 2, 4, and 6, Bond owns the qubits 1, 3, and 7, and Calvin owns the qubit 5, respectively.

**Figure 2:**
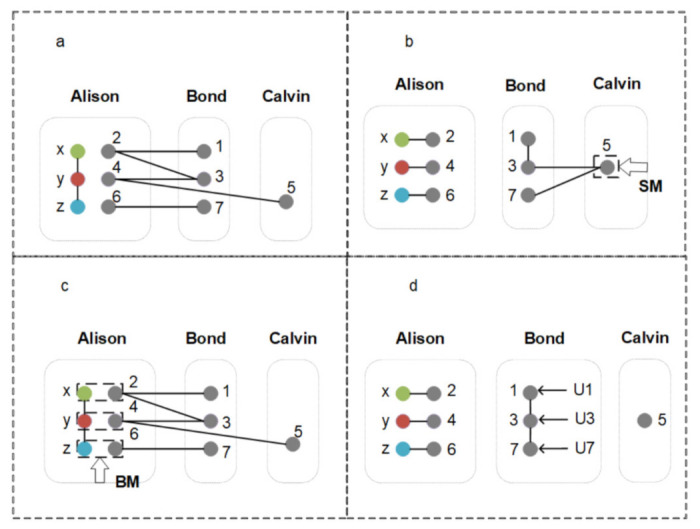
The QIS process between the three parties. The particles connected by solid lines are in an entangled state. BM represents the Bell basis measurement, and SM represents the single-qubit measurement, U1, U3 and U7 represent unitary operations on the particles 1, 3 and 7, respectively. In the step 1 (**a**), Alison, Bond and Calvin possess the particles 246, 137 and 5, respectively. In the step 2 (**b**), Alison performs three times BM on her owned qubit pairs (*x*, 2), (*y*, 4) and (*z*, 6), respectively. In the step 3 (**c**), Calvin performs SM on particle 5. In the step 4 (**d**), Bond reconstructs the state to be transmitted through appropriate U1, U3 and U7 operations.

The principle of our quantum information splitting scheme is shown in Figure 2, in which the particles connected by solid linesare in an entangled state. BM represents the Bell basis measurement, and SM represents the single-qubit measurement; U1, U3, and U7 represent unitary operations on the particles 1, 3, and 7, respectively. Next, the specific process is described in detail.

**Step 1** Share the particles of quantum entangled states securely

Firstly, the sender Alison and the agents Bond and Calvin securely distribute the ${|\psi \rangle }_{12345}$ state and the ${|\phi^{+}\rangle }_{67}$ state. Alison, Bond, and Calvin possess the particles 246, 137, 5, respectively. At the same time, Alison has the three particles of the three-qubit state ${|\psi \rangle }_{xyz}$.

**Step 2** Alison performs the Bell basis measurements

Alison performs the BM three times on her owned qubit pairs (*x*, 2), (*y*, 4), and (*z*, 6), respectively. The Bell basis measurement is $|\phi^{\pm }\rangle \;=\;\frac{1}{\sqrt{2}}\left(|00\rangle \;\pm \;|11\rangle \right)$ and $\;|\varphi^{\pm }\rangle \;=\;\frac{1}{\sqrt{2}}\left(|01\rangle \;\pm \;|10\rangle \right)$. After that, it is possible to obtain 43measurement results in an equal probability form, and she will get one of them. Meanwhile, the residual particles will collapse and acquire one of the corresponding states through the above operation. In the following equations, the symbol ± in $\;\;{|\vartheta \rangle }_{1357}$ represents the results of three qubit pairs (*x*, 2), (*y*, 4), and (*z*, 6) under the Bell basis $\left\{|\phi^{\pm }\rangle ,\;\;|\varphi^{\pm }\rangle \right\}$ in the left-to-right order, respectively. See Equations (11)–(18) for the specific measurement outcomes.

(11) $$\begin{array}{ll} {|\phi^{\pm }\rangle }_{x2}{|\phi^{\pm }\rangle }_{y4}{|\phi^{\pm }\rangle }_{z6}\;{|\vartheta \rangle }_{1357} & =\frac{1}{8}\left(\delta_{000}|0000\rangle ++\pm \delta_{001}|0001\rangle \right.\\ & +\pm +\delta_{010}|0110\rangle +\pm \pm \delta_{011}|0111\rangle \pm ++\delta_{100}|1110\rangle \\ & {\left.\pm +\pm \delta_{101}|1111\rangle \pm \pm +\delta_{110}|1000\rangle \pm \pm \pm \delta_{111}|1001\rangle \right)}_{1357} \end{array}$$

(12) $$\begin{array}{ll} {|\phi^{\pm }\rangle }_{x2}{|\phi^{\pm }\rangle }_{y4}{|\varphi^{\pm }\rangle }_{z6}\;{|\vartheta \rangle }_{1357} & =\frac{1}{8}\left(\delta_{000}|0001\rangle ++\pm \delta_{001}|0000\rangle \right.\\ & +\pm +\delta_{010}|0111\rangle +\pm \pm \delta_{011}|0110\rangle \pm ++\delta_{100}|1111\rangle \\ & {\left.\pm +\pm \delta_{101}|1110\rangle \pm \pm +\delta_{110}|1001\rangle \pm \pm \pm \delta_{111}|1000\rangle \right)}_{1357} \end{array}$$

(13) $$\begin{array}{ll} {|\phi^{\pm }\rangle }_{x2}{|\varphi^{\pm }\rangle }_{y4}{|\phi^{\pm }\rangle }_{z6}\;{|\vartheta \rangle }_{1357} & =\frac{1}{8}\left(\delta_{000}|0110\rangle ++\pm \delta_{001}|0111\rangle \right.\\ & +\pm +\delta_{010}|0000\rangle +\pm \pm \delta_{011}|0001\rangle \pm ++\delta_{100}|1000\rangle \\ & {\left.\pm +\pm \delta_{101}|1001\rangle \pm \pm +\delta_{110}|1110\rangle \pm \pm \pm \delta_{111}|1111\rangle \right)}_{1357} \end{array}$$

(14) $$\begin{array}{ll} {|\phi^{\pm }\rangle }_{x2}{|\varphi^{\pm }\rangle }_{y4}{|\varphi^{\pm }\rangle }_{z6}\;{|\vartheta \rangle }_{1357} & =\frac{1}{8}\left(\delta_{000}|0111\rangle ++\pm \delta_{001}|0110\rangle \right.\\ & +\pm +\delta_{010}|0001\rangle +\pm \pm \delta_{011}|0000\rangle \pm ++\delta_{100}|1001\rangle \\ & {\left.\pm +\pm \delta_{101}|1000\rangle \pm \pm +\delta_{110}|1111\rangle \pm \pm \pm \delta_{111}|1110\rangle \right)}_{1357} \end{array}$$

(15) $$\begin{array}{ll} {|\varphi^{\pm }\rangle }_{x2}{|\phi^{\pm }\rangle }_{y4}{|\phi^{\pm }\rangle }_{z6}\;{|\vartheta \rangle }_{1357} & =\frac{1}{8}\left(\delta_{000}|1110\rangle ++\pm \delta_{001}|1111\rangle \right.\\ & +\pm +\delta_{010}|1000\rangle +\pm \pm \delta_{011}|1001\rangle \pm ++\delta_{100}|0000\rangle \\ & {\left.\pm +\pm \delta_{101}|0001\rangle \pm \pm +\delta_{110}|0110\rangle \pm \pm \pm \delta_{111}|0111\rangle \right)}_{1357} \end{array}$$

(16) $$\begin{array}{ll} {|\varphi^{\pm }\rangle }_{x2}{|\phi^{\pm }\rangle }_{y4}{|\varphi^{\pm }\rangle }_{z6}\;{|\vartheta \rangle }_{1357} & =\frac{1}{8}\left(\delta_{000}|1111\rangle ++\pm \delta_{001}|1110\rangle \right.\\ & +\pm +\delta_{010}|1001\rangle +\pm \pm \delta_{011}|1000\rangle \pm ++\delta_{100}|0001\rangle \\ & {\left.\pm +\pm \delta_{101}|0000\rangle \pm \pm +\delta_{110}|0111\rangle \pm \pm \pm \delta_{111}|0110\rangle \right)}_{1357} \end{array}$$

(17) $$\begin{array}{ll} {|\varphi^{\pm }\rangle }_{x2}{|\varphi^{\pm }\rangle }_{y4}{|\phi^{\pm }\rangle }_{z6}\;{|\vartheta \rangle }_{1357} & =\frac{1}{8}\left(\delta_{000}|1000\rangle ++\pm \delta_{001}|1001\rangle \right.\\ & +\pm +\delta_{010}|1110\rangle +\pm \pm \delta_{011}|1111\rangle \pm ++\delta_{100}|0110\rangle \\ & {\left.\pm +\pm \delta_{101}|0111\rangle \pm \pm +\delta_{110}|0000\rangle \pm \pm \pm \delta_{111}|0001\rangle \right)}_{1357} \end{array}$$

(18) $$\begin{array}{ll} {|\varphi^{\pm }\rangle }_{x2}{|\varphi^{\pm }\rangle }_{y4}{|\varphi^{\pm }\rangle }_{z6}\;{|\vartheta \rangle }_{1357} & =\frac{1}{8}\left(\delta_{000}|1001\rangle ++\pm \delta_{001}|1000\rangle \right.\\ & +\pm +\delta_{010}|1111\rangle +\pm \pm \delta_{011}|1110\rangle \pm ++\delta_{100}|0111\rangle \\ & {\left.\pm +\pm \delta_{101}|0110\rangle \pm \pm +\delta_{110}|0001\rangle \pm \pm \pm \delta_{111}|0000\rangle \right)}_{1357} \end{array}$$

**Step 3** Calvin performs the single-qubit measurement

Alisontransmits the Bell measurement outcomes to Calvin and Bond using the classical channel. Only through Calvin’s assistance can Bond reconstruct the shared information. Consequently, to gain the original information, Calvin performs the *X* basismeasurement toparticle 5 on his own. After that, Calvin will obtain one of the two possible results at random, and the particles (1,3,7) held by Bond will collapse to the corresponding state.

**Step 4** Bond reconstructs the state to be transmitted

Bond recovers this unknown state under the cooperation of Calvin. By combining the outcomes of Alison and Calvin, Bond can carry out aproper unitary operation on the particles he owns to restore the state to be transmitted.

For the sake of a simple but general description of the process, we take Alison’s measurement outcomes ${|\phi^{-}\rangle }_{x2}{|\phi^{-}\rangle }_{y4}{|\phi^{-}\rangle }_{z6}$ as an example to expound and prove the process of the splitting scheme. The remaining particles will collapse into the following state:

(19) $$\begin{array}{ll} {|\vartheta \rangle }_{1357} & =\frac{1}{8}\left(\delta_{000}|0000\rangle -\delta_{001}|0001\rangle -\delta_{010}|0110\rangle +\delta_{011}|0111\rangle \right.-\delta_{100}|1110\rangle \\ & {+\delta_{101}|1111\rangle +\delta_{110}|1000\rangle -\delta_{111}|1001\rangle }_{1357}\\ & =\frac{1}{8\sqrt{2}}\left[{|+\rangle }_{5}\right.\left(\delta_{000}|000\rangle -\delta_{001}|001\rangle -\delta_{010}|010\rangle +\delta_{011}|011\rangle -\delta_{100}|110\rangle \right.\\ & {\left.+\delta_{101}|111\rangle +\delta_{110}|100\rangle -\delta_{111}|101\rangle \right)}_{137}+{|-\rangle }_{5}(\delta_{000}|000\rangle -\delta_{001}|001\rangle \\ & +\delta_{010}|010\rangle -\delta_{011}|011\rangle +\delta_{100}|110\rangle -\delta_{101}|111\rangle +\delta_{110}|100\rangle -\delta_{111}|101\rangle )137] \end{array}$$

If Calvin, the controller, is willing to help Bond, he will make the *X* basis measurement on the particle5he owns, and then he transmits the measurement outcome to Bond. The *X* basis measurement is $|+\rangle =\frac{1}{\sqrt{2}}\left(|0\rangle +|1\rangle \right)$ and $\;|-\rangle =\frac{1}{\sqrt{2}}\left(|0\rangle -|1\rangle \right)$. If the result is ${|+\rangle }_{5}$ or ${|-\rangle }_{5}$, Bond needs to perform the corresponding operation $C_{13}Z_{3}Z_{7}$ or $C_{13}Z_{7}$ on his owned particles 1, 3 and 7, respectively. By completing the procedures mentioned above, Bond can accurately recover the information to be transmitted.

## 4. Security and Efficiency

We analyze the quantum information splitting scheme proposed in this paper from two aspects of security and efficiency in this section. The security is described from external eavesdropper attacks and internal participant attacks. Then, the efficiency of the qubit and the whole scheme is given in turn.

### 4.1. Security Analysis

#### 4.1.1. Internal Participant Attack

Let us first discuss the case of an internal attack. Suppose the controller Calvin is the dishonest agent. He intercepts the Bell measurement results sent by Alison. After that, Calvin sends the usurped entangled information that is prepared in advance to Bond. Therefore, when Bondis responsible for reconstructing the quantum information, he will get the wrong original quantum state. At this time, Bond has a suspicion that there is an insider so that the prepared original quantum state is wrong. When Alison and Bond compare the partial information with the state reconstructed through a classical communication channel, they can detect the existence of an attack. Therefore, the communication is discarded.

#### 4.1.2. External Eavesdropper Attack

An external eavesdropper, Eve, manages to steal the information of Alison, Bond, and Calvin. Eve wants to obtain the state to be transmittedby entangling an auxiliary qubit to the whole system during the particle distribution process. However, it is assumed that the three legitimate parties in the scheme are not conscious of the eavesdropping from Eve. After Alison performs the BM three times, the quantum system of Calvin, Bond, and Eve collapses into a state of entanglement. Then, Calvin performs the *X* basis measurement; at this time, the Bond−Eve system collapses into the tensorproductoftwo states. Eve can not gainany helpful information about the original stateto be transmitted in the process.

Take Alison’s measurement results of ${|\phi^{+}\rangle }_{x2}{|\phi^{+}\rangle }_{y4}{|\phi^{+}\rangle }_{z6}$ as an example. If Eve’s entangled auxiliary particles are $\frac{1}{\sqrt{2}}{\left(|0\rangle +|1\rangle \right)}_{E}$, then the entangled state of Bond, Calvin, and Eve can be expressed as follows:

(20) $$\begin{array}{l} {|\Xi \rangle }_{1357E}\;=\;\frac{1}{8\sqrt{2}}\left(\delta_{000}|00000\rangle +\delta_{000}|00001\rangle +\delta_{001}|00010\rangle +\delta_{001}|00011\rangle \right.\\ \;\;\;\;\;+\delta_{010}|01100\rangle \;+\delta_{010}|01101\rangle +\delta_{011}|01110\rangle +\delta_{011}|01111\rangle \\ \;\;\;\;\;+\delta_{100}|11100\rangle +\delta_{100}|11101\rangle +\delta_{101}|11110\rangle +\delta_{101}|11111\rangle \\ \;\;\;\;\;{\left.+\delta_{110}|10000\rangle +\delta_{110}|10001\rangle +\delta_{111}|10010\rangle +\delta_{111}|10011\rangle \right)}_{1357E} \end{array}$$

If Calvin’s *X* basis measurement outcome is $\left\{{|+\rangle }_{5}\right\}$, then the remaining particles in the Bond−Eve system will collapse into the ${|\xi_{1}\rangle }_{137E}$ state.

(21) $$\begin{array}{l} {|\xi_{1}\rangle }_{137E}\;=\;\frac{1}{16}\left(\delta_{000}|0000\rangle +\delta_{000}|0001\rangle +\delta_{001}|0010\rangle +\delta_{001}|0011\rangle \right.\\ \;\;\;\;\;+\delta_{010}|0100\rangle +\delta_{010}|0101\rangle +\delta_{011}|0110\rangle +\delta_{011}|0111\rangle \\ \;\;\;\;\;+\delta_{100}|1100\rangle +\delta_{100}|1101\rangle +\delta_{101}|1110\rangle +\delta_{101}|1111\rangle \\ \;\;\;\;\;{\left.+\delta_{110}|1000\rangle +\delta_{110}|1001\rangle +\delta_{111}|1010\rangle +\delta_{111}|1011\rangle \right)}_{137E}\\ \;\;\;\;\;=\frac{1}{8\sqrt{2}}\left(\delta_{000}|000\rangle +\delta_{001}|001\rangle +\delta_{010}|010\rangle +\delta_{011}|011\rangle +\delta_{100}|110\rangle \right.\\ \;\;\;\;\;{\left.+\delta_{101}|111\rangle +\delta_{110}|100\rangle +\delta_{111}|101\rangle \right)}_{137}⊗\frac{1}{\sqrt{2}}{\left(|0\rangle +|1\rangle \right)}_{E} \end{array}$$

If Calvin’s *X* basis measurement outcome is $\left\{{|-\rangle }_{5}\right\}$, then the remaining particles in the Bond−Eve system will collapse into the ${|\xi_{2}\rangle }_{137E}$ state.

(22) $$\begin{array}{l} {|\xi_{2}\rangle }_{137E}\;=\;\frac{1}{16}\left(\delta_{000}|0000\rangle +\delta_{000}|0001\rangle +\delta_{001}|0010\rangle +\delta_{001}|0011\rangle \right.\\ \;\;\;\;\;-\delta_{010}|0100\rangle -\delta_{010}|0101\rangle -\delta_{011}|0110\rangle -\delta_{011}|0111\rangle \\ \;\;\;\;\;-\delta_{100}|1100\rangle -\delta_{100}|1101\rangle -\delta_{101}|1110\rangle -\delta_{101}|1111\rangle \\ \;\;\;\;\;{\left.+\delta_{110}|1000\rangle +\delta_{110}|1001\rangle +\delta_{111}|1010\rangle +\delta_{111}|1011\rangle \right)}_{137E}\\ \;\;\;\;\;=\frac{1}{8\sqrt{2}}\left(\delta_{000}|000\rangle +\delta_{001}|001\rangle -\delta_{010}|010\rangle -\delta_{011}|011\rangle -\delta_{100}|110\rangle \right.\\ \;\;\;\;\;{\left.-\delta_{101}|111\rangle +\delta_{110}|100\rangle +\delta_{111}|101\rangle \right)}_{137}⊗\frac{1}{\sqrt{2}}{\left(|0\rangle +|1\rangle \right)}_{E} \end{array}$$

It is easy to see that Eve acquires no information about the original state.

### 4.2. Efficiency Analysis

The security of the QIS process is guaranteed by establishing a secure quantum channel. In distributing particles by the sender, decoy photons technology is introduced to detect the presence of eavesdroppers. The decoy state used to detect eavesdropping accounts for only a small part, which can be neglectedin the abstract. Hence, the qubits’ efficiency $\eta =q_{u}/q_{t}$ can reach 100% in our scheme, where the amount of the useful qubitsis expressed as $q_{u}$, and the amount of qubits transferredis expressed as $q_{t}$. Cabello [35] gave a formula of the efficiency of a quantum key distribution scheme from the perspective of the quantum information theory. Here, the total efficiency of the quantum information splitting scheme can be calculated from the following:

(23) $$\varepsilon \;=\;\frac{q_{s}}{q_{t}\;+\;b_{t}}$$

where $q_{s}$ stands for the quantity of qubits of the quantum state to be shared and $q_{t}$ and $b_{t}$ stand for the quantity of qubits transferred and the total number of classical bits used by agents to transmit the measurement results in the communication process, respectively. In our QIS for sharing an arbitrary three-qubit state between the three parties, the total efficiency is $\varepsilon \;=\;\frac{q_{s}}{q_{t}\;+\;b_{t}}\;=\;\frac{3}{4\;+\;7}\;=\;\frac{3}{11}$, which is the maximum value for the splitting scheme, in theory. Nevertheless, in Yin et al.’s scheme [36] for splitting the quantum state between the three legal parties, the efficiency is $\varepsilon \;=\;\frac{q_{s}}{q_{t}\;+\;b_{t}}\;=\;\frac{3}{5\;+\;8}\;=\;\frac{3}{13}$, which is lower than that of our QIS scheme.

We summarize the comparison of arbitrary three particle states under different quantum channels and give the following Table 1. On the one hand, in comparison to Ref. [32], although the efficiency is the same as ours, it sacrifices more complex quantum resources to recover the quantum state. On the other hand, in comparison to Ref. [36], it is worth noting that we use less quantum resources and improve the efficiency of the scheme.

## 5. Conclusions

To sum up, a novel scheme is studied and proposed about the QIS of an arbitrary three-qubit system via a five-qubit cluster state and a Bell state as the quantum entanglement resource. In this scheme, Alison performs the BM on the owned three pairs of qubits and then sends out the measurement outcomes to Bond and Calvin through a classical communication channel. It is impractical for Bond to recover the secret by performing a unitary operation without the help of Calvin. Consequently, to recover the state to be transmitted, Calvin is supposed to carry out the *X* basis measurement and informs Bond of the measurement outcome with the aid of a classical channel. By the measurement results of Alison and Calvin, Bond can recover the state to be transmitted using an applicable unitary operation on his qubits. In addition, the scheme is demonstrated to be secure under internal and external attacks. Therefore, the scheme is experimentally achievable.

## Figures and Tables

**Table 1:** The comparison for using the different quantum channels to split an arbitrary three-qubit state.

	Entanglement Resource	*q_t_*	*b_t_*	Efficiency
Nie’s scheme [32]	genuinely entangled five-qubit state + Bell state	4	7	3/11
Yin’s scheme [36]	five-qubit cluster state + GHZ state	5	8	3/13
our scheme	five-qubit cluster state + Bell state	4	7	3/11

## Data Availability

Not applicable.
